# Refugees/Immigrants and leishmaniasis in the world’s largest hosting country, Türkiye: A systematic review

**DOI:** 10.1371/journal.pntd.0012947

**Published:** 2025-04-07

**Authors:** Nermin Şakru, Yusuf Özbel, Seray Töz

**Affiliations:** 1 Department of Medical Microbiology, University of Trakya, School of Medicine, Edirne, Türkiye; 2 Department of Medical Parasitology, University of Ege, School of Medicine, İzmir, Türkiye; Hospital Universitário Professor Edgard Santos, Brazil

## Abstract

**Background:**

This systematic literature analysis discusses cutaneous leishmaniasis (CL) and visceral leishmaniasis (VL) in Türkiye, emphasizing the increasing importance of species determination studies and epidemiological research due to the emergence of new causative agents post-2010. It highlights the influx of immigrants and refugees into Türkiye, particularly from the neighboring countries where conflict occur.

**Materials and methods:**

The study conducted a systematic review of research on leishmaniasis among refugees/immigrants in Türkiye between 2010 and 2022. A search in literature was carried out via English (PubMed, Scopus and Web of Science) and Turkish (TRDizin, and Council of Higher Education Thesis Center) databases published between 1 January 2010 and 31 December 2022. Two reviewers assessed the full-text articles to determine final eligibility.

**Results:**

A total of 1,356 studies were identified, and 20 studies for CL, two studies for CL/VL and one study for VL (23 studies, 25 data set) were included in this systematic review. In these 22 studies, a total of 4,337 positive CL cases were reported. Among these positives 1,381 cases were diagnosed in Turkish individuals. The remaining 2,956 positive CL cases were the immigrants from different countries including Syria (n=2,925), Iraq (n=13), Afghanistan (n=7), Somalia (n=4), Iran (n=3) and one case each of Libya, Turkmenistan, Tunisia and Morocco. We also identified the presence of 94 positive visceral leishmaniasis cases, with 75 cases being among Turkish individuals and 19 cases among Syrian refugees in three studies.

**Conclusions:**

The systematic review underscores the significance of international epidemiological data sharing and robust health monitoring systems to manage the global public health threat posed by leishmaniasis and other infectious diseases related to population movements.

## Author summary

The frequency of leishmaniasis, a group of diseases caused by sand flies, is influenced by various factors such as climate change, environmental factors, living conditions, individual characteristics, and large-scale human movements. In this systematic review, leishmaniasis cases detected and published in immigrants coming to Turkey for various reasons, primarily due to the Syrian civil war, one of the countries with the highest number of foreign migrants, have been compiled.

In this review, articles including immigrant patients were chosen covering over four thousand leishmaniasis cases have been documented, with 70% of these cases being migrants. This finding contributes to the creation of data that can be used in various fields. Additionally, it should not be overlooked that patients who have been diagnosed and treated may also be present in areas outside of the centers where the publications were conducted.

The arrival of patients with the same disease from abroad to a country where suitable vector species exist and where the disease is already present increases the risk of transmission from a public health perspective. It is believed that combining and analyzing studies conducted on this specific group of patients is important for providing epidemiological data and to see the picture above on this vector-borne disease.

## Introduction

Türkiye is the world’s largest hosting country for migrants and refugees, especially from neighboring regions in conflict like Syria, Iraq, Afghanistan, and also the main land route to get into Europe for the refugees from Bhutan, Myanmar, Bangladesh, India, Pakistan and Nepal [1,2]. Moreover, smaller groups of refugees and immigrants from various other nations have also settled in Türkiye, further contributing to the overall migrant population in the country. As of the statement issued in November 2023, it was reported that the number of regular immigrants stood at 4,643,986, among which 3,237,585 were Syrians [3].

Two clinical forms of leishmaniasis, cutaneous and visceral, have historically existed in Türkiye for a very long time. The disease is transmitted by vector sand flies, and suitable climatic and geographical conditions, along with locations fostering microclimates conducive to sand fly breeding, ensure the continuity of these diseases. In Türkiye, it was previously known that *Leishmania tropica (L. tropica)* is the main causative agent of cutaneous leishmaniasis (CL), while *Leishmania infantum (L. infantum)* is responsible for both cutaneous and visceral leishmaniasis (VL) [4,5]. However, after 2010, with the increase in international migration, the detection of two other *Leishmania* species *(L. major and L. donovani)* in the Old World as causative agents of cutaneous leishmaniasis has emphasized the importance of species typing studies in samples taken from patients and in epidemiological studies in Türkiye [6–9].

Because of an important immigrant population residing in Türkiye, in this study, it was aimed to draw attention to the issue by analyzing the data in published studies examining the leishmaniasis situation among refugees/immigrants in Türkiye.

## Materials and methods

### Search strategy

This systematic study was conducted according to the Preferred Reporting Items for Systematic Reviews and Meta-Analysis (PRISMA) guidelines [10]. This review has considered all original studies including cohort, cross-sectional, retrospective, case reports and theses conducted on immigrants related to human leishmaniasis in Türkiye. This search in literature was carried out via English [PubMed, Scopus and Web of Science (WoS)] and Turkish (TRDizin, and Council of Higher Education Thesis Center) databases published between 1 January 2010 and 31 December 2022. The search was conducted from May to July of 2023. The present study employed terms including: “cutaneous leishmaniasis”, “visceral leishmaniasis”, “Leishman*”, “Kala azar”, “Şark çıbanı [Turkish]”, and “Türkiye” alone or in combination with “OR” and/or “AND”, in Turkish and English.

### Study selection and data extraction

The criteria for inclusion of a study were as follows: (a) studies conducted in Türkiye, (b) availability of full-text articles, and (c) inclusion of at least one diagnostic method such as microscopy, culture, serology, or molecular techniques. Exclusion criteria encompassed: (a) review studies, book chapters, (b) duplicated data, (c) articles lacking full-text accessibility, (d) studies associated with animal leishmaniasis, and (e) cases designated by authors as imported or originating from foreign sources.

This literature search was conducted using the PubMed, Scopus, and Web of Science (WoS) databases and all records exported to EndNote v20.5. Once the duplicates were removed, they were exported to Excel. The Turkish literature and theses were similarly transferred to Excel, and all the searched studies were then consolidated.

We reviewed articles, removing those that did not meet the inclusion criteria. The “refugee”, “immigrant”, “Syria” and “Syrian” words have been scanned in the titles, abstracts and keywords of the articles. Records that appeared potentially eligible after primary screening were selected to download the full text. Two reviewers assessed the full-text articles to determine final eligibility, resolving any contradictions among studies through discussion and consensus. One author extracted the required data, which was then cross-checked by a second author. Additionally, the reference lists of selected full-text papers were manually examined to identify articles not retrieved by the database search.

The extracted data from the literature included: first author, year of publication, geographical region of the study, types of clinical manifestations, sample size, number of examined cases, number of positive cases, and diagnostic laboratory tests.

## Results

**Fig 1:**
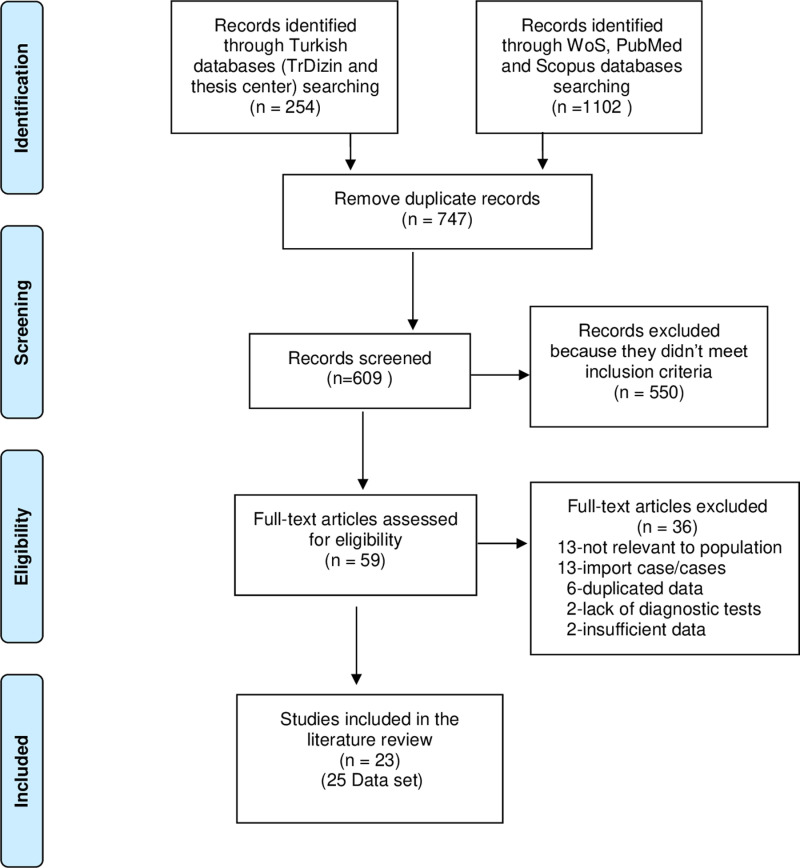
Flow diagram of selected articles used for present systematic review.

A total of 1,356 studies were identified, consisting of 1,102 from WoS, PubMed, and Scopus databases, and 254 from Turkish databases (TRdizin and National Thesis Center). Initially, 747 duplicated studies were removed, leaving 609 studies for evaluation. Among these, 550 did not meet the inclusion criteria. From the remaining 59 full-text articles, 36 were excluded due to reasons such as insufficient data, unclear information regarding the number of refugees/immigrants, or containing imported cases. 20 studies for CL, two studies for CL/VL and one study for VL were included in this review. Consequently, a total of 23 studies (25 data set) were included in this systematic review. (Fig 1).

In total, the 23 studies enrolled 5,721 suspected leishmaniasis cases, comprising 5,577 CL cases and 144 VL suspected cases. Among the 23 studies only three were focused on immigrant cases of VL. These three studies collectively identified 94 positive VL cases, with 75 cases being among local Turkish individuals and 19 cases among Syrians.

**Fig 2:**
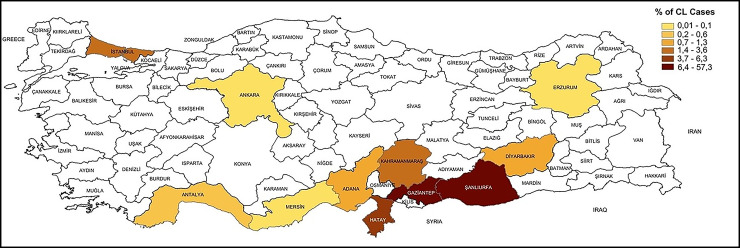
Türkiye map showing distribution of CL cases based-on province level reported in the selected published papers. (The map is prepared using ArcGIS software and the source of base layer of the map is Republic of Türkiye Ministry of National Defence, Directorate General for Mapping. Available from https://www.harita.gov.tr/urunler/downloadable-products/13 (free).

In contrast, the majority of studies were concerned with immigrant cases of CL and a total of 4,337 positive CL cases were reported. Between these positives 1,381 cases were identified among local Turkish individuals. The remaining 2,956 positive CL cases were the immigrants from different countries including Syria (n=2,925), Iraq (n=13), Afghanistan (n=7), Somalia (n=4), Iran (n=3) and one case each from Tunisia, Morocco, Turkmenistan, and Libya (Table 1). The distribution of CL cases across provinces, based on data from selected published research, is shown on a map of Türkiye (Fig 2).

**Table 1:** Characteristics of the studies including in the systematic literature analysis.

	Reference (by published year)	Studied year	Disease form	F	M	Province studied	Diagnostic method	Total smear positive	Total population number (n)	Turkish	Syrian	Other Countries	Subtype of CL	Most common age range (yrs)
1	Kocarslan et al., 2013 [11]	2012-2013	CL	22	32	Şanlıurfa	Mic&Hist	54	54	30	24	–	Noduloulcerative (57%) and Papulonodular (33%)	0-20 (67%)
2	Turan et al., 2014 [12]	na	CL	0	2	Şanlıurfa	Mic	2	2	1	1	–	Papular	na
3	Salman et al., 2014 [13]	2010-2013	CL	47	30	Gaziantep	Mic	77	416	15	62	–	na	0-19 (68%)
4	Turhanoglu et al., 2014 [14]	2005-2013	CL	41	15	Diyarbakır	Mic	56	128	47	9	–	Papular and ulcerated	0-20
5A	Koltaş et al., 2014 [6]	2003-2013	CL	na	na	Adana, Şanlıurfa, Hatay, Gaziantep, Kahramanmaraş, Diyarbakır	Mol	107	167	93	14	–	na	na
6	Inci et al., 2015 [15]	2011-2014	CL	60	50	Kahramanmaraş	Mic&Hist	110	110	34	76	–	Noduloulcerative (54%) and Papulonodular (43%)	0-20 (53%)
7A	Aydın Teke et al., 2015 [16]	na	CL	0	1	Ankara	Mic	1	1	–	1	–	Ulcerated	3
8	Turan et al., 2015 [17]	2012	CL	742	628	Şanlıurfa	Mic&Hist	1370	1370	685	685	–	Ulcerated (48%) and Nodular (49%)	0-15
9	Demirkan et al., 2015 [18]	2014-2015	CL	101	77	Birecik	Mic&Hist	178	178	42	136	–	na	1-20 (78%)
10	Korkmaz et al., 2015 [19]	2013-2014	CL	299	336	Gaziantep	Mic&Hist	635	635	67	568	–	na	0-20 (66%)
11	Ozkeklikci et al., 2017 [20]	2009-2015	CL	129	134	Gaziantep	Mic&Mol	263	567	88	174	1	na	0-20 (54%)
12	Karakuş et al., 2019 [21]	2014-2018	CL	na	na	İstanbul	Mic&Mol	25	25	0	25	–	na	na
13	Eroglu & Özgöztaşı, 2019 [22]	2013-2015	CL	482	418	Gaziantep	Mic	900	1100	55	845	–	Papular and Nodular	0-20 (68%)
14	Kaya et al., 2020 [23]	2015-2018	CL	47	74	Hatay	Mic	121	121	34	87	–	Papular (67%), Nodular (11%), Plaque 14%) and Ulcerated (7%)	0-18 (100%)
15	Kurt et al., 2020 [24]	2006-2018	CL	12	21	Erzurum	Hist	33	40	27	2	4	na	na
16	Yazisiz et al., 2020 [25]	2010-2019	CL	98	97	Antalya	Mic	37	195	17	19	1	na	na
17	Yıldız Zeyrek et al., 2020 [26]	2016	CL	5	9	Şanlıurfa	Mic&Mol	14	14	10	4	–	na	na
18	Serarslan et al., 2020 [27]	2017-2019	CL	33	51	Hatay	Mol	84	84	16	68	–	na	na
19	Yentur Doni et al., 2020 [28]	2015-2017	CL	69	85	Şanlıurfa	Mic&Mol	154	196	94	60	–	Ulcerated (51%), Nodular (44%) and Papular (5%)	na
20	Alshekissa A., 2021 [29]	2019-2021	CL	8	8	Mersin	Mic&Mol	6	16	1	5	–	na	na
21	Sirekbasan & Polat, 2021 [30]	2002-2017	CL	15	14	İstanbul	Mic	29	77	22	4	3	na	na
22	Altinel & Taş, 2022 [31]	2010-2017	CL	32	49	İstanbul	Mic	81	81	3	56	22	Crusted papular (97%)	0-18 (46%)
**TOTAL**								**4337**	**5577**	**1381**	**2925**	**31**		
5B	Koltaş et al., 2014 [6]	2003-2013	VL	na	na	Adana, Hatay, Şanlıurfa, Gaziantep, Kahramanmaraş, Diyarbakır	Mol	63	113	51	12	–	–	
7B	AydınTeke et al., 2015 [16]	na	VL	0	1	Ankara	Mic	1	1	–	1	–	–	
23	Alabaz et al., 2022 [32]	na	VL	10	20	Adana, Hatay, Gaziantep, Osmaniye, Adıyaman	Mic&, Mol & Rapid Test	30	30	24	6	–	–	
**TOTAL**								**94**	**144**	**75**	**19**		–	

**CL**: Cunateous leishmaniasis; **VL**: Visceral leishmaniasis; **Mic**: Microscopy; **Hist**: Histopathology; **Mol**: Molecular; **na**: not available; **F**: Female; **M**: Male; **Other countries**: Iraq, Somali, Iran, Tunisia, Morocco, Afghanistan, Turkmenistan, Libya

Seven studies utilized molecular techniques for diagnosing and identifying *Leishmania* species in the cases of CL, while two studies employed similar techniques for VL. These studies targeted gene regions such as ITS1 and kDNA. Identification of species was achieved through various methods, including analyzing melting curves in real-time PCRs (RT-PCR), and employing Restriction Fragment Length Polymorphism (RFLP) or sequencing subsequent to conventional PCRs in different research investigations. Within the CL samples across six studies utilizing species identification techniques, a total of 409 positive cases were identified. Among these cases, 176 were immigrants. Specifically, 149 cases were identified as *L. tropica*, 5 as *L. infantum*, and 22 as *L. major* among immigrant populations. Regarding the VL samples in two studies employing species identification techniques, a total of 90 positive cases were detected, with 18 among immigrants. Among these, 13 cases were identified as *L. donovani*, 4 as *L. infantum*, and 1 as *L. tropica* within immigrant groups (Table 2).

**Table 2:** Studies that molecular analyses were conducted.

References	PCR Type	Gene region	Total Positive	*L. infantum*		*L. tropica*		*L. major*		*L. donovani*	
				Local	Imm	Local	Imm	Local	Imm	Local	Imm
**CL**											
Koltas et al., 2014 [6]	RT-PCR&Sequencing	kDNA&ITS1	107	39	–	45	–	9	14	–	–
Özkeklikçi et al., 2017 [20]	RT-PCR	ITS-1	20	1	1	13	5	–	–	–	–
Karakuş et al., 2019 [21]	RT-PCR (MLMT)	ITS-1	24	–	–	–	24	–	–	–	–
Yıldız Zeyrek et al., 2020 [26]	RT-PCR	ITS-1	14	4	1	6	2	–	1	–	–
Seraslan et al., 2020 [27]	PCR-RFLP	ITS-1	84	11	1	5	67	–	–	–	–
Yentur Doni et al., 2020 [28]	PCR-RFLP	ITS-1	154	–	2	89	51	5	7	–	–
Alshekissa A., 2021 [29]	Conventional	Miniexon	6	–	–	–	–	–	–	–	–
**TOTAL**			**409**	**55**	**5**	**158**	**149**	**14**	**22**	**–**	**–**
**VL**											
Koltaş et al., 2014 [6]	RT-PCR&Sequencing	kDNA&ITS1	63	38	–	6	–	–	–	7	12
Alabaz et al., 2022 [32]	RT-PCR, PCR-RFLP& Sequencing	kDNA&ITS1	27	8	4	6	1	–	–	7	1
**TOTAL**			**90**	**46**	**4**	**12**	**1**	**–**	**–**	**14**	**13**

**Imm**: Immigrants

## Discussion

At the end of May 2023, 110 million people, of which 35.3 million were refugees, forcibly displaced by persecution, conflict, violence and human rights violations. The Syrian crisis has forced millions of Syrians to flee, making them the largest group of refugees with 6.5 million hosted in 131 countries. More than 75 percent are hosted in neighbouring countries such as Türkiye (3.5 million), Lebanon (814,700) and Jordan (660,900) [1].

In Türkiye, the onset of the Syrian civil war in 2011 significantly accelerated mass migration from Syria to Türkiye. Furthermore, ongoing migration from Afghanistan, albeit in smaller groups, has notably added to the influx of migrants arriving in Türkiye from these two countries, both of which have a high incidence of CL. Consequently, this surge in migration led to a substantial increase in the recorded cases of CL in 2013 and 2014, totalling over 5000 cases when combined with local cases [33,34].

Türkiye, given its proximity to Syria and the ongoing civil war, has predominantly received immigrants from Syria. Cutaneous leishmaniasis remains a noteworthy public health concern in the geographical region shared by Türkiye and Syria. Mass migration worldwide has evolved into a global issue, impacting not only humanitarian concerns but also contributing to the spread of diseases across countries and continents. Diseases transmitted by arthropod vectors can easily disseminate to geographically neighbouring recipient countries, as observed between Syria and Türkiye, Iraq, and Lebanon [35–37].

Alvar et al. conducted a study to investigate the global incidence of CL between 2004 and 2011. In the Mediterranean Basin, comprising 26 countries, the study reported the highest case numbers in Algeria (44,050) followed by Syria (22,882) from 2004 to 2008. Furthermore, during the same period, Syria reported 14 cases of VL [38]. An epidemiological study conducted in Syria, highlighted a concerning increase in CL cases, rising from 17,709 in 2007 to 82,275 in 2018 [39]. Cutaneous leishmaniasis in Syria continue to rise, with the last official figure reporting 79 327 confirmed cases in 2021 [40].

The articles chosen for this systematic review indicate that the initial study concerning leishmaniasis in immigrants was published in 2013, focusing on refugees residing in camps situated in the south eastern region. Subsequently, the number of related studies gradually increased, totalling 59 publications by 2022. However, our analysis could only include 23 studies that met the established inclusion criteria. These selected studies have underscored the significant role of migration in the spread of vector-borne diseases.

The majority of the studies included in the analysis used parasitological diagnosis in addition to molecular and histopathological techniques. Serological methods, known for their low sensitivity in diagnosing CL, are limitedly available in Turkish laboratories, even for VL, therefore none of the studies in our analysis employed serological methods for CL cases. Among the eight studies reporting CL cases using molecular techniques, the ITS1 gene region was predominantly targeted. Additionally, one study each targeted the miniexon and kDNA+ITS1 gene regions. Conversely, molecular techniques focusing on the kDNA+ITS1 gene regions were employed in two studies reporting VL cases among immigrants. Moreover, in three studies (one CL and two VL), the PCR products from the kDNA and ITS1 regions underwent sequencing procedures.

A total of 1,069 suspected cases were examined using PCR techniques targeting various gene regions and 409 cases (6 cases were not identified in species level) were positive. Among immigrants, three *Leishmania* species were identified in 176 CL cases (16,46%) out of the total suspected cases. Among these 176 CL cases, the most prevalent species was *L. tropica* (149 cases; 84,65%), followed by *L. major* (22 cases; 12,50%), and lastly, *L. infantum* (5 cases; 2,84%). Similar results were observed among the Turkish population (227 cases), with *L. tropica* being the most prevalent species (158 cases; 69,60%). Following *L. tropica, L. infantum* was the second most abundant species (55 cases; 24,22%), while *L. major* was the least detected species (14 cases; 6,16%) (Table 2). Apart from these three species, previous studies on CL have indicated the presence of *L. donovani* as a causative agent in various regions of Türkiye [8].

Species identification using molecular techniques was conducted in two out of three studies involving VL cases. A total of 143 suspected cases underwent PCR analysis targeting kDNA and ITS1 regions, resulting in the identification of three *Leishmania* species. Among the 143 suspected cases, 90 tested positive. Of these positive cases, 18 were immigrants, with the species distribution as follows: four cases of *L. infantum* (4.44%), one case of *L. tropica* (1.11%), and 13 cases of *L. donovani* (14.44%). Regarding the remaining 72 positive cases among the Turkish population, the species identification was distributed as follows: 46 cases of *L. infantum* (51.11%), 12 cases of *L. tropica* (13.33%), and 14 cases of *L. donovani* (15.55%). In a single study, MLMT (Multi-Locus Microsatellite Typing) analysis was conducted on 24 samples of *L. tropica* obtained from immigrant patients with CL admitted to hospitals from 2014 to 2018. Population structure analysis was carried out by incorporating samples from Turkish and Syrian patients obtained from earlier studies. This particular study revealed the presence of three primary populations among the *L. tropica* samples. One of these populations consisted solely of Turkish samples, while the remaining two populations comprised a mixture of Syrian and Turkish samples [21]. Previous studies related to VL have also reported the presence of these three species in Türkiye [6,8].

The studies included in the analysis encompassed a broad age range, indicating that cases of the disease can occur across all age groups, from children to the elderly. However, in 11 studies that the ages of the patients have evaluated showing that between 0–20 years is the most affected age group among immigrants. Moreover, the sex ratio among the cases included in all studies was nearly equal, with a ratio of 1.04 (females to males).

Clinical picture of CL patients can vary according to different species of *Leishmania*, the duration of the lesion and lifestyle and immune status of the patient. For these reasons, it is often the case that patients included in the studies, especially in large groups, have very different clinical views [41]. Similarly, in the present literature review, almost all analysed and reported studies have revealed different clinical pictures. Among 22 studies, clinical situation of the CL patients was mentioned only in 10 studies and most common clinical forms were observed as nodulo-ulcerative/ulcerative, papular and nodular, respectively.

Through our literature review in this study, we identified that the majority of suspected cases of CL and VL across various studies were predominantly from Syria. Smaller numbers of cases were reported from Iraq, Somalia, Iran, Tunisia, Morocco, Afghanistan, Turkmenistan, and Libya. This emphasizes the significant impact and importance of the Syrian population in these studies (Table 1). Ten out of the 12 provinces included in the study are situated in the South-eastern or Eastern Regions of Türkiye. These regions have been home to the majority of immigrants residing in refugee camps or independent rented houses since the onset of the civil war in Syria. Once again, epidemiological reports reaffirm that leishmaniasis poses a global public health threat. It emphasizes the crucial need for international collaborative studies and the enhancement of the World Health Organization’s (WHO) recording system to effectively control and manage the number of cases.

The present study had certain limitations. First, regrettably, detailed information regarding their travel route and whether the infection was acquired in Turkey or in foreign countries was unavailable. Consequently, cases classified as ‘imported’ could not be included in our analysis. Second, the study protocol was not registered in PROSPERO because of overloaded with COVID-19 reviews.

Considering Türkiye’s strategic geographical location, we anticipate continued and potentially increased migration. As such, we advocate for the importance of maintaining stringent health controls at points of entry into the country and establishing comprehensive records of immigrants’ existing health conditions. It is imperative to institutionalize and advance the management of migration information, establishing dedicated sections within the notification system for immigrants. This approach will not only foster advancements in the healthcare system but also facilitate prompt and efficient diagnosis and treatment of immigrants. Simultaneously, such measures will serve to prevent the heightened spread of diseases within the country due to these population movements. These insights are not only pertinent to leishmaniasis but also hold relevance for other infectious diseases.

## Supporting information

S1 Table PRISMA (2020) abstract checklist. (DOCX)

S2 Table List of studies excluded at full-text screening and exclusion reasons. (DOCX)

S1 Data Raw data. (XLSX)
