# Supplementary material for: Refugees/Immigrants and leishmaniasis in the world’s largest hosting country, Türkiye: A systematic review
Source: PLoS Negl Trop Dis. 2025 Apr 7;19(4):e0012947. doi: 10.1371/journal.pntd.0012947 (PMC12005488; doi:10.1371/journal.pntd.0012947)
Supplement: S2 Table — (DOCX) [file pntd.0012947.s002.docx]

**S2 Table. List of studies excluded at full-text screening and exclusion reasons**

|  | **Studies** | **Reason for exclusion** |
| --- | --- | --- |
| 1 | Yazar, S., Salih, K. U. K., Cetinkaya, U., & Sahin, I. (2013). Leishmania sp. in cutaneous leishmaniasis suspected patients is Kayseri. Ankara Üniversitesi Veteriner Fakültesi Dergisi, 60(3), 177-178. | Import cases |
| 2 | Guven Gokmen T. (2013) Comparison of Sensitivity and Utility of Mini-exon-PCR-RFLP and ITS-PCRRFLP Methods Used to Diagnosis and Determining of Agent Types inLeishmaniasis. PhD thesis. | Import cases |
| 3 | Zeyrek, F. Y., Gürses, G., Uluca, N., Doni, N. Y., Toprak, Ş., Yeşilova, Y., & Çulha, G. (2014). Is the agent of Cutaneous Leishmaniasis in Sanliurfa changing? First cases of Leishmania major. Turkiye Parazitol Derg, 38, 270-4. | Import case |
| 4 | Çulha, G., Doğramacı, Ç. A., Gülkan, B., & Savaş, N. (2014). Cutaneous leishmaniasis and its status in Hatay province, Turkey. Turkish Bulletin of Hygiene and Experimental Biology, 71(4), 171-178. | Not relevant to population |
| 5 | Kartal, D., Çınar, S. L., Akın, S., Ferahbaş, A., & Borlu, M. (2015). Skin findings of geriatric patients in Turkey: A 5-year survey. Dermatologica sinica, 33(4), 196-200. | Not relevant to population |
| 6 | Ekiz, Ö., Rifaioǧlu, E. N., Şen, B. B., Çulha, G., Özgür, T., & Doǧramaci, A. Ç. (2015). Leishmaniasis recidiva cutis of the lips mimicking granulomatous cheilitis. Indian Journal of Dermatology, 60(2), 216. | Import case |
| 7 | Duman, R., Duman, N., Yavaș, G. F., Doğan, M., & Duman, R. (2015). Lesson of the month 2: Chronic erythematous painless plaque on the eyelid co-presenting with multiple ulcerated nodules on the extremities. Clinical Medicine, 15(3), 304. | Import case |
| 8 | Yeşilova, Y., Turan, E., Sürücü, H. A., Aksoy, M., & Özbilgin, A. (2015). Successful Treatment of Cutaneous Leishmaniasis with Amphotericin B; A Case of Unresponsive to Pentavalent Antimony Therapy. Turkiye Parazitol Derg, 39, 63-5. | Not relevant to population |
| 9 | Aksoy, M., Doni, N., Ozkul, H. U., Yesilova, Y., Ardic, N., Yesilova, A., ... & Satoskar, A. R. (2016). Pediatric cutaneous leishmaniasis in an endemic region in Turkey: a retrospective analysis of 8786 cases during 1998-2014. PLoS neglected tropical diseases, 10(7), e0004835. | Not relevant to population |
| 10 | İnci R, Ozturk P, Mülayim MK, et al: Dermatological face of Syrian civil war. Arch Turk Dermatol Venerology 2016;50:145-9. | Duplicated data |
| 11 | Kaman, A., Tanır, G., Gayretli Aydın, Z. E. Y. N. E. P., Karlı Oğuz, H. A. T. İ. C. E., Metin, Ö., Aydın Teke,.. & Mungan, M. (2018). Cutaneous leishmaniasis in pediatric patients in a single tertiary hospital in Ankara. Turkish Journal of Parasitology, 41(4). | Import cases |
| 12 | ZEYREK, F. Y., TÖZ, S., YÜKSEL, F., TURGAY, N., & ÖZBEL, Y. (2017). Comparison of Polymerase Chain Reaction Using Kinetoplast DNA Specific Primers and Other Parasitological Methods in the Diagnosis of Clinical Samples of Suspected Patients with Cutaneous Leishmaniasis in Şanlıurfa. Mikrobiyol Bul, 51(4), 340-349. | Not relevant to population |
| 13 | Karakuş, M., Nasereddin, A., Onay, H., Karaca, E., Özkeklikçi, A., Jaffe, C. L., ... & Töz, S. (2017). Epidemiological analysis of Leishmania tropica strains and giemsa-stained smears from Syrian and Turkish leishmaniasis patients using multilocus microsatellite typing (MLMT). PLoS neglected tropical diseases, 11(4), e0005538. | Insufficent data |
| 14 | Şener, S., Karaman, Ü., Hakverdi, G., & Saraç, G. (2017). Evaluation of Patients with Cutaneous Leishmaniasis Who Admitted to Dermatology Clinic in Kahramanmaras Sutcu Imam University Medical Faculty KONURALP TIP DERGİSİ.2017-Cilt: 9 - Sayı: 3 274-277. | Not relevant to population |
| 15 | Beyhan, Y. E., Karakus, M., Karagoz, A., Mungan, M., Ozkan, A. T., & Hokelek, M. (2017). Detection and identification of cutaneous leishmaniasis isolates by culture, polymerase chain reaction and sequence analyses in Syrian and Central Anatolia patients. Saudi Medical Journal, 38(9), 968. | Import cases |
| 16 | TARTAR, A. S., BALIN, Ş. Ö., & AKBULUT, A. (2018). Is The Dıstrıbutıon Of Gerıatrıc Infectıons Dıfferent In Eastern Turkey? Retrospectıve Evaluatıon Of Gerıatrıc Infectıons. Turkish Journal Of Geriatrics, 21(2). | Not relevant to population |
| 17 | Gurses, G., Ozaslan, M., Zeyrek, F. Y., Kılıç, I. H., Doni, N. Y., Karagöz, I. D., & Uluca, N. (2018). Molecular identification of Leishmania spp. isolates causes cutaneous leishmaniasis (CL) in Sanliurfa Province, Turkey, where CL is highly endemic. Folia microbiologica, 63, 353-359. | Duplicated data |
| 18 | ÇULHA, G., DOĞRAMACI, A. Ç., KAYA, T., ÇAVUŞ, İ., GÜLKAN, B., & ÖZBİLGİN, A. (2018).Imported Cutaneous Leishmaniasis Cases Detected in Truck Drivers in Hatay. Mikrobiyoloji Bülteni, 52(3), 316-323. | Import cases |
| 19 | Sirekbasan, S., & Polat, E. (2018). Real-time PCR using high-resolution melting analysis technology for diagnosis of Leishmania and determination of types of clinical samples. Turkish Journal of Medical Sciences, 48(6), 1358-1363. | Import cases |
| 20 | Özbilgin, A., Töz, S., Harman, M., Topal, S. G., Uzun, S., Okudan, F., ... & Özbel, Y. (2019). The current clinical and geographical situation of cutaneous leishmaniasis based on species identification in Turkey. Acta tropica, 190, 59-67. | Not relevant to population |
| 21 | Karaosmanoğlu, N., Şahin, M., Vahaboğlu, G., Akbay, G., Edgüer, E. Y., Şahin, T., ... & Adiloğlu, A. K. (2019). Cutaneous leishmaniasis: evaluation of 117 Syrian immigrants. Türkiye Klinikleri. Tip Bilimleri Dergisi, 39(2), 160-164. | Import cases |
| 22 | Çizmeci, Z., Karakuş, M., Karabela, Ş. N., Erdoğan, B., & Güleç, N. (2019). Leishmaniasis in Istanbul; A new epidemiological data about refugee leishmaniasis. Acta tropica, 195, 23-27. | Duplicated data |
| 23 | Özbilgin, A., Gencoglan, G., Tunali, V., Çavuş, İ., Yıldırım, A., Gündüz, C., & Harman, M. (2020). Refugees at the crossroads of continents: a molecular approach for cutaneous leishmaniasis among refugees in Turkey. Acta Parasitologica, 65, 136-143. | Import cases |
| 24 | Çulha, G., DoĞramaci, A. Ç., Hakverdi, S., SeÇİntİ, İ. E., AslantaŞ, Ö., Çelİk, E., & Tuğba, K. A. Y. A. (2020). The investigation of the association of cutaneous leishmaniasis in biopsy specimens of the patients with granulomatous disease and skin cancer using the molecular method. Iranian Journal of Parasitology, 15(3), 307. | Insufficent data |
| 25 | Bayram G, Dinçer E, Erden Ertürk S, Tiftik EN. Seroprevalence of asymptomatic Leishmania spp. carriage among blood donors in leishmaniasis endemic area in Turkey. FLORA 2020;25(1):33-9. | Not relevant to population |
| 26 | Çulha, G., Kaya, T., & Dogramaci, A. Ç. (2020). Genotyping of Cutaneous Leishmaniasis Cases Detected Before and After Migration with Real-Time Polymerase Chain Reaction in Hatay. Turkish Journal of Parasitology, 44(1), 48-52. | Import cases |
| 27 | Çabalak, M., Çulha, G., Bal, T., Kaya, T., & Çelik, E. (2021). Cutaneous Leishmaniasis with Mucosal Involvement. Türkiye Parazitoloji Dergisi, 45(3), 227. | Import cases |
| 28 | Yakşi N, İkiişik H, Güner Ae, Maral I. clınıcal and epıdemıologıcal characterıstıcs of cutaneous leıshmanıasıs cases in istanbul. ESTÜDAM Halk Sağlığı Dergisi. 2021;6(3):209-15. | Lack of diagnostic tests |
| 29 | Fettahlioglu Karaman, B., Aksungur, V., Unal, I., & Uzun, S. (2022). Factors Associated with Early Referral for Pediatric Cutaneous Leishmaniasis. Acta Dermatovenerologica Croatica, 30(2), 89-93. | Not relevant to population |
| 30 | Gürses, G., & Yiğin, A. (2022). Typing of Leishmania Species Causing Cutaneous Leishmaniasis by Sybr Green Based ITS-1 Real Time Polymerase Chain Reaction Method. *Mikrobiyoloji Bulteni*, *56*(2), 326-338. | Not relevant to population |
| 31 | İnal, N., Altıntop, T. Ü., Ergüven, S., & Yılmaz, Y. A. (2022). Retrospective Results of Hacettepe University Faculty of Medicine Parasitology Laboratory Between 2014-2019. Turkiye Parazitol Derg, 46(2), 114-8. | Not relevant to population |
| 32 | Cay, U., Alabaz, D., Uguz, A. H., & Yanar, H. (2022). Etiology of granulomatous inflammation: A retrospective study of 174 children in a tertiary care center. Asian Pacific Journal of Tropical Medicine, 15(11), 511-517. | Not relevant to population |
| 33 | Bener, F. (2020). Analysis of dermatological diseases of Syrian asylum seekers. *TURKDERM-Turkish Archives of Dermatology and Venereology*, *54*(3), 96-102 | lack of diagnostic tests |
| 34 | Kirecci E, Ozturk P, Guler S, Gul M, Karakas T, Timur D. Retrospective evaluation of the patients diagnosed with cutaneous leishmaniasis during the period of 2011-2013 in Kahramanmaras Province. Mersin Univ Saglik Bilim Derg. 2013;6(2):16-9. | Duplicated data |
| 35 | Polat E, Kutlubay Z, Sirekbasan S. Treatment of Glucantime-resistant/ tolerant cutaneous leishmaniasis with Lucilia sericata larvae and its larval secretions: The first study in the world. Trop Biomed. 2016;33(4):668-74. | Duplicated data |
| 36 | Ekşi F, Özgöztaşı O, Karslıgil T, Sağlam M. Genotyping Leishmania promastigotes isolated from patients with cutaneous leishmaniasis in south-eastern Turkey. J Int Med Res. 2017;45(1):114-22. doi: 10.1177/0300060516677155. | Duplicated data |
